# The Effect of the Referral System on the Accessibility of Healthcare Services: A Case Study of the Wuhan Metropolitan Development Zone

**DOI:** 10.3390/ijerph191610441

**Published:** 2022-08-22

**Authors:** Ying Chen, Jiale Wu

**Affiliations:** College of Public Administration, Huazhong University of Science and Technology, Wuhan 430074, China

**Keywords:** referral system, public healthcare, geographical accessibility, equity of healthcare, Wuhan Metropolitan Development Zone

## Abstract

The geographical accessibility of public healthcare institutions is the key factor affecting the equity of healthcare services. Based on the hierarchical medical system and referral system in China, we analyzed the referral accessibility of hospitals in the Wuhan Metropolitan Development Zone. Before the implementation of the referral system, only 7.91% of the total communities met the accessibility standard for secondary and tertiary hospitals, which meant that there was significant inequality in high-level healthcare. Moreover, 5.4% of the total communities did not meet the accessibility standard for primary hospitals, which meant that there were insufficient primary hospitals. After the implementation of the referral system, the proportions of communities meeting the accessibility standards for the first-stage referral, second-stage referral and cross-level referral were 92.6%, 99.9% and 98.3%, respectively. The results show that the referral system has improved the accessibility of healthcare, but it has not completely solved healthcare inequality. The first-stage referral accessibility of healthcare services in the northern, western and eastern groups does not meet the accessibility standard, which is due to the inefficient layout of secondary hospitals. The Wuhan government should construct secondary hospitals in these groups and primary hospitals in the central urban area and the southeastern, southern, western and eastern groups.

## 1. Introduction

Healthcare equity refers to the rational distribution of healthcare resources among populations or regions, emphasizing the matching of healthcare resources and social needs in quantity and space. Promoting the rational distribution of healthcare resources is of great significance to human health and sustainable development [1]. China is the largest developing country in the world. Its huge population is a great challenge in the efficient use of public healthcare resources. The data for 2018 show that China’s public hospitals provided more than 85.8% of general outpatient and inpatient healthcare services [2], and this is still increasing. In other words, as the main providers of healthcare services, the number and distribution of public hospitals greatly affect the healthcare equity of the Chinese people. Since its reform and opening, although China’s economy has developed very rapidly, the inequality of healthcare resources has increased. For this reason, the Chinese government is carrying out a series of medical and health system reforms [3,4,5].

In order to achieve healthcare equality for all, the Chinese government has established a hierarchical medical system [6]. This stipulates three levels of healthcare service institutions and a referral system, which enables the hierarchical medical system to play a significant role in achieving healthcare equality. The referral system requires patients to first be diagnosed by primary healthcare service institutions, and then the doctors decide whether to transfer them. The more developed the city is, the more efficiently the hierarchical medical system may operate, and the better the accessibility of health services may be [7]. This is because developed cities perform better than less developed cities in many aspects, such as in terms of social governance and GDP. However, the fact is that healthcare services equity in big cities has not kept pace with urban expansion [8,9]. Since the 21st century, the Chinese government and scholars have paid more attention to the differences in the spatial accessibility of healthcare service resources [10,11] and have also taken spatial accessibility as an important principle for the layout of public service institutions [12,13]. Public healthcare institutions are an important part of public service institutions, and the referral system is influenced by the spatial behavior of patients based on traffic conditions, which includes people going to the hospital from a residential area and the hospital transferring patients to another hospital. Therefore, accessibility is an effective evaluation criterion for a referral system.

Penchansky and Thomas have claimed that accessibility includes five dimensions: availability, accessibility, affordability, acceptability and adaptability [14]. These dimensions are reflected in the use of public healthcare service institutions, such as whether people can physically reach the hospitals and receive treatment, whether they can afford healthcare expenses and whether they can easily move around in the hospitals [15,16,17]. These are the manifestations of different dimensions of “accessibility” [18,19,20]. Geographical accessibility refers to the degree of difficulty in spatial communication between the starting point and the end point [21,22]; it is the geographical basis for exploring the location and spatial layout of public service institutions [23]. With the development of GIS technology, the spatial data of the demand for and supply of healthcare can be used as the basis for evaluating the spatial fairness of health services [24,25,26]. It has become a research paradigm to calculate geographical accessibility through mature mathematical models, including the proportional method, nearest distance method, two-step floating catchment area method (2SFCA) and the potential model [27,28].

In modern society, the individual economic conditions of residents, in addition to the information dissemination effect, influence their choice to go directly to secondary and tertiary hospitals [29], but people’s choice of healthcare services largely depends on traffic conditions [30]. When families own cars, it is still difficult to access healthcare services in remote areas [31]. This means that public transport has a great impact on the spatial accessibility of healthcare services [32]. The low accessibility of public healthcare services is due to inadequate infrastructure construction [33,34,35]. As one of the central cities of China, Wuhan implemented the referral system earlier than others, and its public healthcare resources are concentrated in the Metropolitan Development Zone. Whether the distribution of hospitals at all levels is balanced and whether the referral system can improve the accessibility of healthcare services are important issues. At present, there are very few simulation studies on the referral system. Most of the studies on the accessibility of medical services in a single stage are repetitive and do not identify new problems. Therefore, it is necessary to discuss the changes in the accessibility of public healthcare services before and after the implementation of the referral system in the Wuhan Metropolitan Development Zone because this will clarify the actual effect of the referral system and allow us to put forward countermeasures and suggestions to promote healthcare equity.

The structure of this paper is as follows. Section 2 includes an introduction to China’s hierarchical medical system and referral system. Section 3 describes the study area, data sources and methodology used in this study. Section 4 includes the analysis of the accessibility of healthcare services before and after the referral system. Section 5 presents the discussion. The final section contains the conclusions of this study.

## 2. The Hierarchical Medical System and Referral System in China

**Figure 1:**
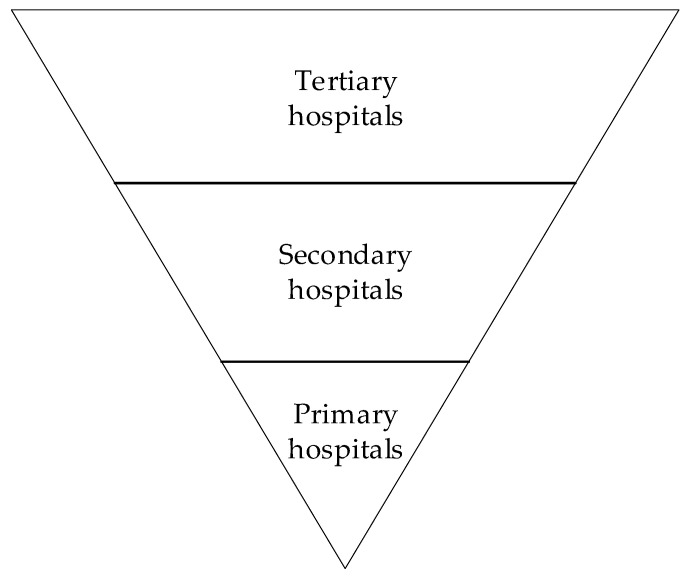
Healthcare resources with inverted pyramid structure.

As early as the period of its planned economy, China had a strict hierarchical medical system. However, the reform and opening up of its economy has led to the gradual disintegration of this system. Due to the increase in income and the development of transportation, the original method of adjusting for the location of patients through price differences has gradually failed [36,37]. This has led to more and more people preferring to go directly to secondary and tertiary hospitals. In 2009, the Chinese government promised to provide equal basic healthcare services to all citizens [38]. In 2015, the government resolved to establish the hierarchical medical system by 2020 and therefore made the construction of a hierarchical medical system the primary task of medical reform in the 13th Five-Year Plan. A hierarchical medical system means that different types of patients should be treated at different levels of healthcare institutions, which are divided into tertiary, secondary and primary hospitals [39]. According to the data of *China Statistical Yearbook of Health and Family Planning 2021*, the number of secondary and tertiary hospitals with equipment occupation of more than CNY 10,000 is 6.93 times the total number of primary hospitals, and the number of beds is 3.17 times the total number of beds in primary hospitals. Secondary and tertiary hospitals hold a large number of high-end healthcare resources, while primary hospitals are facing a simultaneous shortage of equipment and doctors [40]. Therefore, the medical system is polarized, and the limited public healthcare resources are wasted [41]. This makes the public health system an inverted pyramid structure (Figure 1).

Wuhan’s public healthcare resources are greater than the Chinese average. The tertiary hospitals in Wuhan include four national scientific research hospitals, namely Union Hospital, Tongji Hospital, Zhongnan Hospital of Wuhan University and Hubei General hospital. Because these four hospitals are affiliated with Huazhong University of Science and Technology and Wuhan University, they not only undertake the important task of treating patients from all over the country but also important medical research. The secondary hospitals in Wuhan undertake the task of treating patients from the whole city and even the whole province. Primary hospitals are set up according to the geographical division of the community, mainly for the treatment of basic diseases.

The key to the efficient operation of the hierarchical medical system is the implementation of the referral system [42]. China’s referral system is a bottom-up model, and referral from secondary and tertiary hospitals to primary hospitals is rare [43,44]. It should be noted that the accessibility of patients to primary hospitals for rehabilitation treatment after discharge is not within the scope of this paper.

**Figure 2:**
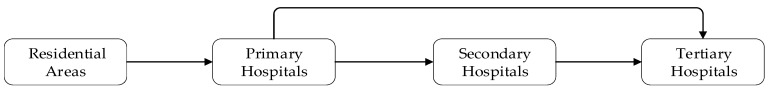
Process of the referral system.

Under this system, when people need healthcare assistance, the government encourages them to go to primary hospitals. When primary hospitals are unable to treat them, they need to be referred to secondary or tertiary hospitals (Figure 2). Therefore, in this study we discuss and simulate the referrals from primary hospitals to secondary or tertiary hospitals.

## 3. Materials and Methods

### 3.1. Study Area and Data

**Figure 3:**
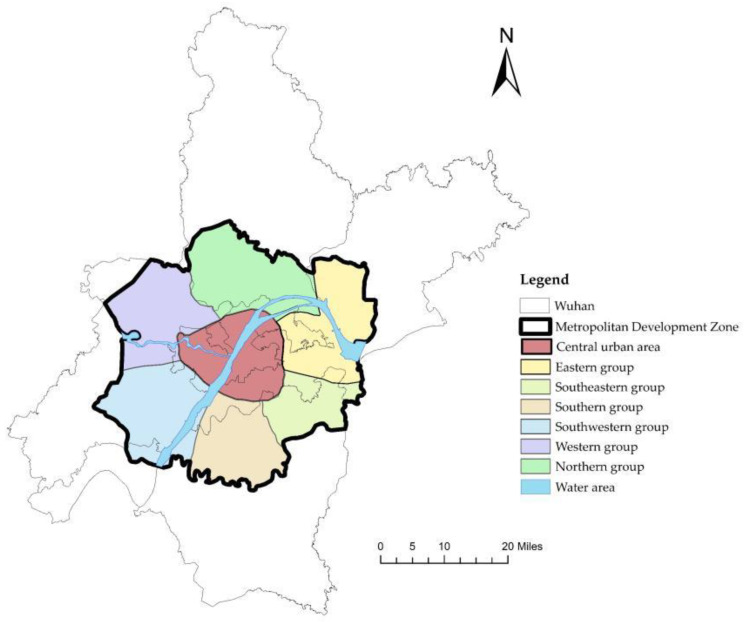
Wuhan Metropolitan Development Zone.

The study area was the Wuhan Metropolitan Development Zone, defined in *Wuhan New-type Urbanization Planning (2014–2020)*, with an area of 3271.59 km^2^, accounting for 37% of the area of Wuhan (Figure 3). Wuhan can be divided into the Metropolitan Development Zone and the Ecological Agriculture Zone, and the tasks undertaken by the two are quite different. The Wuhan Metropolitan Development Zone mainly undertakes the tasks of public service construction and economic construction, and it includes about 85% of the population and 90% of the public medical facilities of Wuhan. The Wuhan Metropolitan Development Zone consists of the central urban area and six urban groups. Due to the spatial distribution of the Yangtze River and the Han River, the Wuhan central urban area is divided into three parts: Wuchang, Hankou and Hanyang. In 2016, Wuhan began to implement the referral system. The goal was essentially to build a high-quality and efficient healthcare service system by 2020. In recent years, some hospitals have been added to the urban development areas. However, what is the current state of the healthcare service system and the referral system in the Wuhan Metropolitan Development Zone? We evaluated them by simulating the referral system and calculating accessibility.

**Figure 4:**
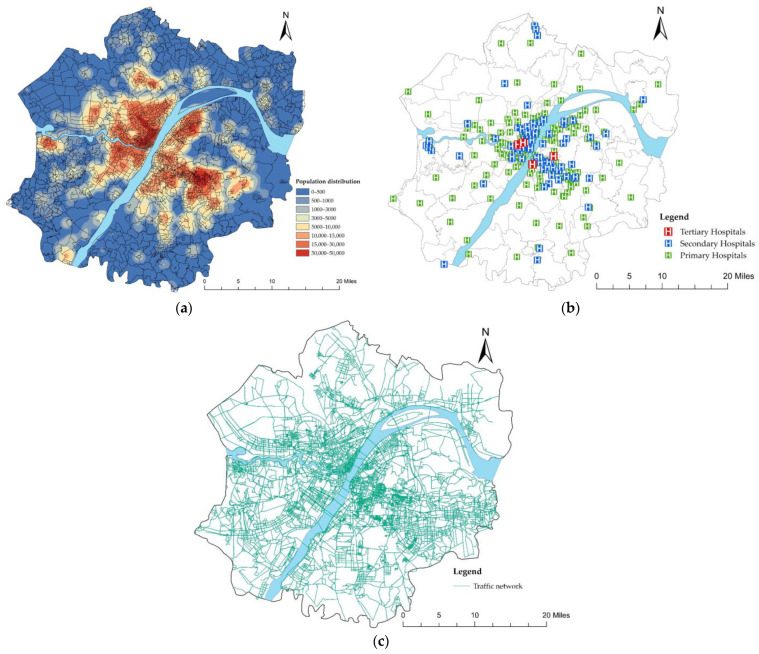
(**a**) Distribution of population in the Wuhan Metropolitan Development Zone; (**b**) distribution of hospitals in the Wuhan Metropolitan Development Zone; (**c**) traffic network in the Wuhan Metropolitan Development Zone.

This study took a residential area as the research unit, including both urban and rural residential areas. We captured the data of the residential areas using Python and retained 5192 items after eliminating invalid data. According to the census data of Wuhan, the population of the residential areas was counted according to the standard of 2.47 people per household (Figure 4a).

Hospital data were obtained through government websites and Baidu maps. We obtained data for 4 tertiary hospitals, 79 secondary hospitals and 161 primary hospitals, and the distribution of hospitals is shown in Figure 4b. According to the hospitals’ official websites and their government documents, the number of beds in Hubei General Hospital, Zhongnan Hospital of Wuhan University, Tongji Hospital and Union Hospital was 3500, 3300, 4000 and 4600, respectively. The number of beds in secondary hospitals was 200–2500. The number of beds in primary hospitals was 30. The open resource data were provided by the road network for OpenStreetMap (Figure 4c). After we obtained the traffic network data through the OpenStreetMap website, any irrelevant roads, such as steps, were eliminated to facilitate the simulation of the actual situation.

### 3.2. Method

#### 3.2.1. Potential Model

The potential model is derived from the gravity model. The nearest distance method neglects the quantity and quality of the research units, while the proportion method cannot reflect the spatial barrier effect of the supply and demand sides. Compared with these two methods, the potential model can comprehensively consider such factors as the spatial barrier and the distance attenuation between hospitals and residential areas [45,46]. Therefore, this model can more comprehensively reflect the accessibility level. The formulae for evaluating the accessibility level are as follows:

(1) $$A_{i}=\sum_{j=1}^{n}\frac{M_{j}}{D_{ij}^{\beta }V_{j}}$$

(2) $$V_{j}=\sum_{k=1}^{m}\frac{P_{k}}{D_{kj}^{\beta }}$$

where $A_{i}$ refers to the spatial accessibility from the residential area i to all public healthcare institutions; $M_{j}$ represents the service capacity of the public healthcare institutions (the number of hospital beds is adopted in this study); $D_{ij}$ and $D_{kj}$ refer to the traffic impedance (time or distance) from the residential area to the public healthcare institutions; $V_{j}$ refers to the sum of the influencing factors of the public healthcare institutions j on the population size of all accessible settlements, that is, the common use or consumption of the service capacity of the public healthcare institutions j by different settlements; β is the friction coefficient representing traffic impedance; n and m represent the number of hospitals and residents, respectively; and $P_{k}$ represents the population of settlement k.

Most studies value β in the range of 1–2, and some will first compare the calculation results when β is equal to 1 or 2, respectively, and then select the value of β according to the degree of conformity between the calculation results and the actual situation [47,48,49,50]. Therefore, we first calculated the accessibility of β with values of 1 and 2 and then selected the appropriate value for the subsequent calculation.

#### 3.2.2. Setting of Parameters and Standards

**Figure 5:**
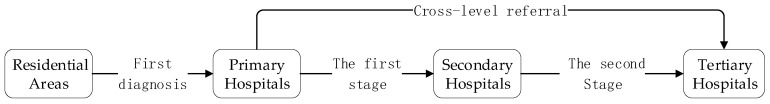
Different stages of referral.

We divided the referral system into three stages: the first stage, the second stage and cross-level referrals. The first stage refers to the transition from primary hospitals to secondary hospitals. The second stage refers to the transition from secondary hospitals to tertiary hospitals. A cross-level referral means a direct transfer from primary hospitals to tertiary hospitals (Figure 5). When the referral system was not implemented, we calculated that, according to the hospital beds per 1000 persons published in *Statistical Yearbook of Wuhan* and *Basic Standards of Chinese Primary Hospitals*, the accessibility standard value of secondary and tertiary hospitals was 6.5 and that of primary hospitals was 1. Since the Chinese government has not set the standard value of referral accessibility, in this paper it needed to be simulated and calculated according to the actual situation. In accordance with the service capacity of hospitals at different levels and the standardized community population, we set an ideal situation—that is, when the demand point of referrals can fully enjoy the healthcare service ability of the end point—and then calculated the standard value of healthcare referral accessibility at each stage using the potential model: the first stage was 20; the second stage was 3; and the cross-level referral was 50. These standard values were used to determine whether the referral accessibility at each stage was up to standard.

When the referral system was not implemented, the residential areas were the demand point of healthcare services. When calculating the accessibility of referrals in different stages, primary or secondary hospitals needed to be regarded as the demand point.

The average speed limits of urban trunk roads, secondary trunk roads and branch roads in the Wuhan urban development area are 60 km/h, 40 km/h and 30 km/h, respectively. There are more than 4 million cars in the urban development area; traffic jams are frequent and vehicles are usually unable to drive at the full speed of the speed limit. Therefore, we set the running speed of trunk roads, secondary trunk roads and branch roads as 50 km/h, 40 km/h and 30 km/h, respectively. Moreover, a reasonable limit on travel times is a necessary condition for accessibility analysis in order to more truly reflect the accessibility of different referral stages. According to the residents’ treatment habits and the records of referrals between hospitals, we set the travel time limits as follows: 15 min for the first diagnosis; 30 min for the first and second stage referrals; and 60 min for cross-level referrals.

## 4. Result

### 4.1. Healthcare Services Accessibility before the Referral System

#### 4.1.1. Accessibility of Secondary and Tertiary Hospitals

**Figure 6:**
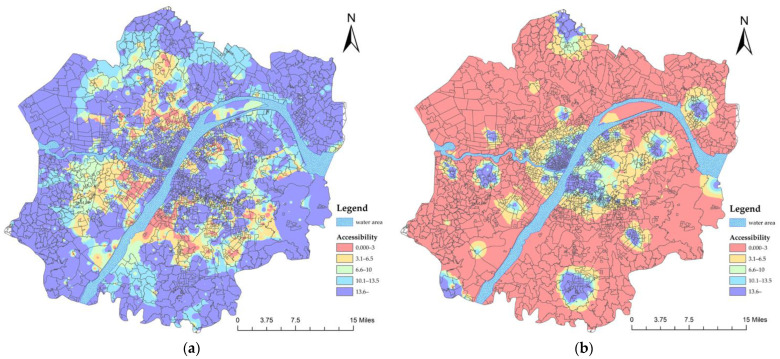
(**a**) Accessibility of secondary and tertiary hospitals (β=1); (**b**) accessibility of secondary and tertiary hospitals (β=2 ).

When the value of the friction coefficient β is 1, the calculation results show that the accessibility distribution is irregular (Figure 6a). The accessibility of the periphery of the Wuhan Metropolitan Development Zone reaches a high value, which is inconsistent with reality. Of course, when the value of the friction coefficient β is 2, the calculation result is in line with reality (Figure 6b). Therefore, in the subsequent accessibility analysis, we kept the value of β as 2.

Before the implementation of the referral system, the accessibility of public healthcare services in the Wuhan Metropolitan Development Zone was generally low. Only 154 communities reached the accessibility standard while 1793 did not (Table 1). High accessibility only existed in the central urban area and near the secondary and tertiary hospitals scattered in the periphery of the Metropolitan Development Zone, which meant that, while secondary and tertiary healthcare services could be obtained easily in the central urban area, it was difficult to enjoy such benefits in the periphery of the Metropolitan Development Zone.

#### 4.1.2. Accessibility of Primary Hospitals

**Figure 7:**
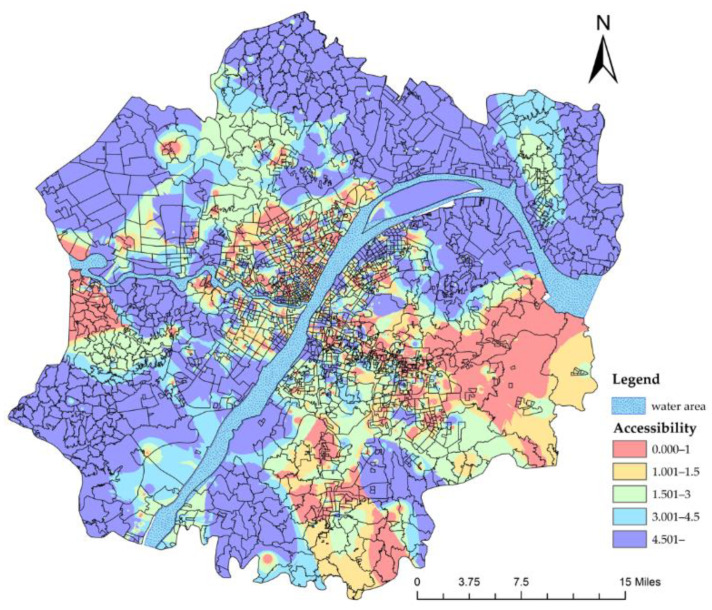
Accessibility of primary hospitals (first diagnosis).

The results show that there are regional differences in the accessibility of primary healthcare services (Figure 7). The accessibility of primary healthcare in some areas of the central urban area, southeastern group, southern group, western group and eastern group did not meet the accessibility standard, including 104 communities, which accounted for 5.4% of the total (Table 2). In other words, in the first diagnosis stage, there has been inequality in primary health care in the Wuhan Metropolitan Development Zone.

### 4.2. Health Services Accessibility after the Referral Reform

**Figure 8:**
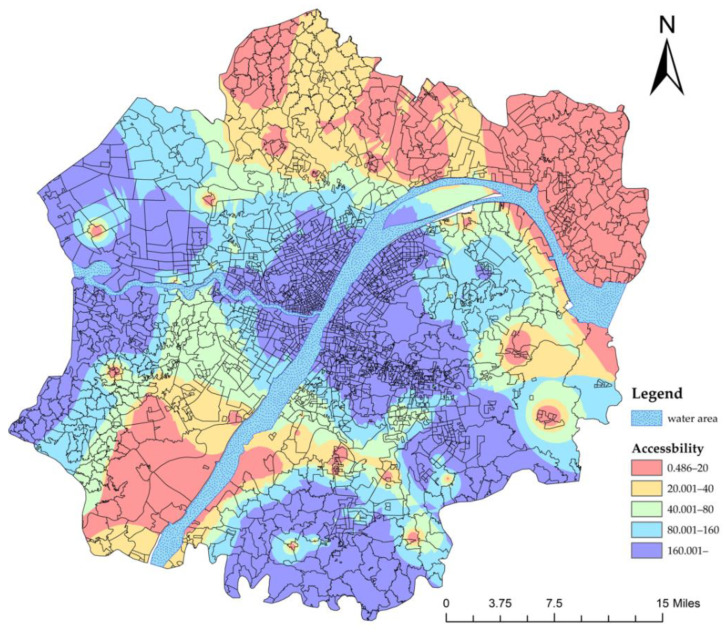
Accessibility of the first stage referral.

The accessibility results of the first-stage referral are shown in Figure 8. We found that the accessibility of secondary healthcare services has been greatly improved by the operation of the referral system. In particular, the accessibility of healthcare services in the western group, southern group, southeastern group and central urban area has reached a high value. In other words, as long as residents in these areas reach the primary hospitals, they do not have to worry about the subsequent diagnosis and treatment. However, the accessibility is very poor in the northern, southwestern and eastern groups. In these areas, 144 communities did not meet the standard (Table 3), which means people living in these areas do not have such benefits.

**Figure 9:**
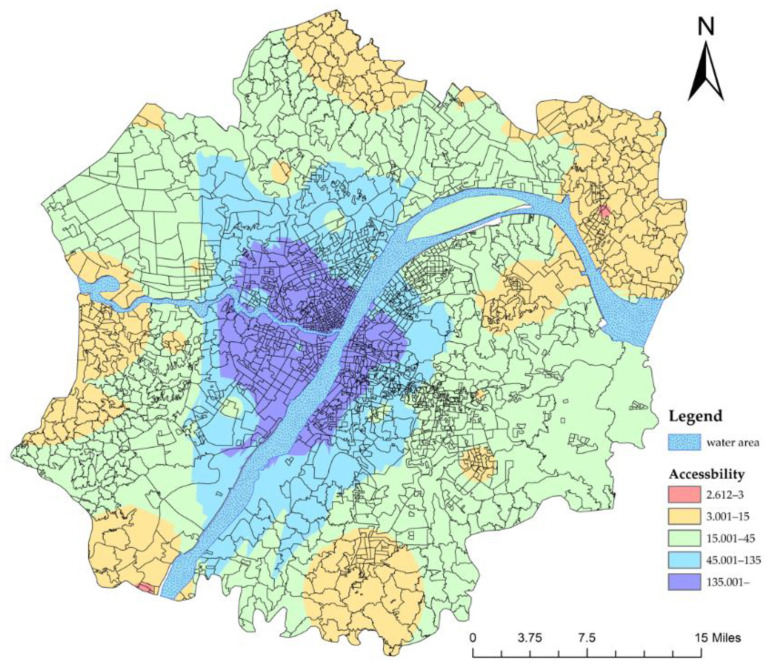
Accessibility of the second-stage referral.

The accessibility results of the second stage of referral are shown in Figure 9. Compared to the first stage of referral, only two communities failed to meet the standard for the accessibility of referrals in the second stage (Table 4). This means that patients who enter or are transferred to any secondary hospital can be transferred to the tertiary hospitals within a reasonable time.

**Figure 10:**
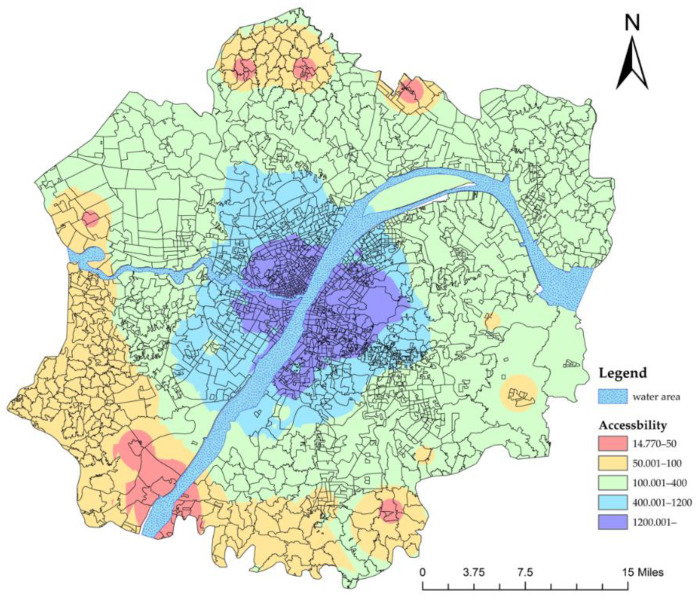
Accessibility of the cross-level referral.

The accessibility results of the cross-level referral are shown in Figure 10. Only 33 communities failed to meet the standard for cross-level referral accessibility, which was concentrated in the southwestern and northern groups (Table 5).

In general, the referral system plays a positive role in increasing the accessibility of public healthcare in the Wuhan Metropolitan Development Zone. Compared with the referral accessibility of the first stage, that of the second stage and cross-level are clearly better.

## 5. Discussion

Before the implementation of the referral system, the residents often went directly to secondary or tertiary hospitals, which directly caused these high-level hospitals to be full of patients, thereby reducing their accessibility. However, after a first diagnosis in a primary hospital, the patients are transferred to the corresponding level of hospital for treatment according to the severity of their disease, which is conducive to the rational allocation of healthcare resources. This is the fundamental reason for the implementation of the referral system in China. According to our research results, 5.4% of the total communities did not meet the accessibility standard of primary hospitals, and they were distributed in the central urban area and the southeastern, southern, western and eastern groups. Therefore, the Wuhan government should increase primary hospitals in the above areas.

After the implementation of the referral system, the accessibility of healthcare services in the Wuhan Metropolitan Development Zone has improved. Although the second-stage and cross-level referral accessibility is satisfactory, the accessibility of the first-stage referral is not as good as we expected. In the first-stage referral, the accessibility is very poor in the northern, southwestern and eastern groups.

There are similarities and differences between the results of this study and other studies. In London, there is inequality in the access to general practitioner services for the elderly, which is mainly caused by the opening hours of hospitals [51]. In Vancouver, Seattle and Portland, there is significant inequality in the access to healthcare services for vulnerable groups, which is caused by the compactness of cities and traffic conditions [52]. The case of Mumbai shows that the income gap and imperfect transportation are the key factors in the inequality of access to healthcare services [53]. For China, despite the great popularity of public transport, there are still regional inequalities in the access to healthcare services. In Changsha, Nanjing, the accessibility of secondary and tertiary healthcare in the central urban area is good, while the peripheral areas are poor [54,55]. The case of Hefei shows that the large-scale flow of patients may bring unnecessary healthcare service costs, thus aggravating healthcare service inequality, which provides strong support for the implementation of the referral system [56]. The research on the accessibility of referrals in Beijing also shows that the referral system can significantly improve the accessibility of healthcare services [42]. In general, healthcare inequality is a common phenomenon around the world, and the causes of inequality are different. The accessibility of referrals in the Wuhan Metropolitan Development Zone is not affected by the terrain because the river-crossing bridge significantly improves the connectivity between different urban centers. However, other multi-center cities may not have the same characteristics [57].

Based on the actual situation in the Wuhan Metropolitan Development Zone, and through the comparison and analysis of the results of many studies, we believe that the inefficient layout of secondary hospitals is the fundamental reason why the first-stage referral accessibility of healthcare services in the northern, western and eastern groups do not meet the accessibility standard. Within these three groups, secondary hospitals and primary hospitals are far away from each other, making it difficult to achieve efficient referral. The secondary hospitals not only take on patients transferred from the primary hospitals but also transfer seriously ill patients to the tertiary hospitals. Therefore, the Wuhan government should construct secondary hospitals in the northern, southwestern and eastern groups.

There are some deficiencies in our research. Firstly, we calculated the demand for healthcare services directly according to the community population and did not distinguish by age, yet the elderly, children and other groups have significantly higher demand for healthcare services than the young and middle-aged groups. Secondly, we did not distinguish between specialized hospitals and general hospitals. Referral should not only consider accessibility but also disease types and their corresponding specialized hospitals, which were not considered in this study. Finally, we simulated the referral system with equal opportunities, without considering the affiliation of and cooperation between different hospitals. If this relationship is reasonably quantified and incorporated into the reachability calculation, the research results will be more accurate.

## 6. Conclusions

Our research took the Wuhan Metropolitan Development Zone as an example and simulated the accessibility of public healthcare services before and after the implementation of the referral system using the potential model. We found that before the implementation of the referral system, there was significant inequality in secondary and tertiary healthcare services, and there were insufficient primary hospitals. After the implementation of the referral system, the accessibility of healthcare services was significantly improved. However, the first-stage referral accessibility of the northern, southwestern and eastern groups did not meet the standard, which is due to the inefficient layout of secondary hospitals. In the next construction of hospitals, the Wuhan government should construct secondary hospitals in the northern, southwestern and eastern groups and construct primary hospitals in the central urban area and the southeastern, southern, western and eastern groups.

## Figures and Tables

**Table 1:** Accessibility of secondary and tertiary hospitals without the referral system.

Accessibility (Standard Value = 6.5)	Number of Communities	Proportion
Up to the standard	154	7.91%
Not up to the standard	1793	92.09%

**Table 2:** Number of communities meeting the standard (first diagnosis).

Accessibility (Standard Value = 1)	Number of Communities	Proportion
Up to the standard	1833	94.6%
Not up to the standard	104	5.4%

**Table 3:** Number of communities meeting the standard (the first stage).

Accessibility (Standard Value = 20)	Number of Communities	Proportion
Up to the standard	1803	92.6%
Not up to the standard	144	7.4%

**Table 4:** Number of communities meeting the standard (the second stage).

Accessibility (Standard Value = 3)	Number of Communities	Proportion
Up to the standard	1945	99.9%
Not up to the standard	2	0.1%

**Table 5:** Number of communities meeting the standard (cross-level referral).

Accessibility (Standard Value = 50)	Number of Communities	Proportion
Up to the standard	1926	98.3%
Not up to the standard	33	1.7%

## Data Availability

Not applicable.
